# Novel Collagen Membrane Formulations with Irinotecan or Minocycline for Potential Application in Brain Cancer

**DOI:** 10.3390/ma17143510

**Published:** 2024-07-15

**Authors:** Andreea-Anamaria Idu, Mădălina Georgiana Albu Kaya, Ileana Rău, Nicoleta Radu, Cristina-Elena Dinu-Pîrvu, Mihaela Violeta Ghica

**Affiliations:** 1 Department of General Chemistry, Faculty of Chemical Engineering and Biotechnologies, National University of Science and Technology POLITEHNICA Bucharest, 011061 Bucharest, Romania; anamaria.idu@gmail.com; 2 Department of Neurosurgery, “Carol Davila” University of Medicine and Pharmacy, 020021 Bucharest, Romania; 3 Collagen Department, INCDTP—Division Leather and Footwear Research Institute, 93 Ion Minulescu Str., 031215 Bucharest, Romania; albu_mada@yahoo.com; 4 Faculty of Biotechnology, University of Agronomic Sciences and Veterinary Medicine of Bucharest Romania, 59 Bulevardul Marasti, 011464 Bucharest, Romania; nicoleta.radu@biotehnologii.usamv.ro; 5 Biotechnology Department, National Institute of Chemistry and Petrochemistry R&D of Bucharest, 202 Splaiul Independentei, 060021 Bucharest, Romania; 6 Department of Physical and Colloidal Chemistry, Faculty of Pharmacy, “Carol Davila” University of Medicine and Pharmacy, 6 Traian Vuia Str., 020956 Bucharest, Romania; cristina.dinu@umfcd.ro (C.-E.D.-P.); mihaela.ghica@umfcd.ro (M.V.G.); 7 Innovative Therapeutic Structures R&D Center (InnoTher), “Carol Davila” University of Medicine and Pharmacy, 6 Traian Vuia Str., 020956 Bucharest, Romania

**Keywords:** nanotechnology, bioengineering, glioblastoma, irinotecan, minocycline, collagen-based membranes

## Abstract

Our study explores the development of collagen membranes with integrated minocycline or irinotecan, targeting applications in tissue engineering and drug delivery systems. Type I collagen, extracted from bovine skin using advanced fibril-forming technology, was crosslinked with glutaraldehyde to create membranes. These membranes incorporated minocycline, an antibiotic, or irinotecan, a chemotherapeutic agent, in various concentrations. The membranes, varying in drug concentration, were studied by water absorption and enzymatic degradation tests, demonstrating a degree of permeability. We emphasize the advantages of local drug delivery for treating high-grade gliomas, highlighting the targeted approach’s efficacy in reducing systemic adverse effects and enhancing drug bioavailability at the tumor site. The utilization of collagen membranes is proposed as a viable method for local drug delivery. Irinotecan’s mechanism, a topoisomerase I inhibitor, and minocycline’s broad antibacterial spectrum and inhibition of glial cell-induced membrane degradation are discussed. We critically examine the challenges posed by the systemic administration of chemotherapeutic agents, mainly due to the blood–brain barrier’s restrictive nature, advocating for local delivery methods as a more effective alternative for glioblastoma treatment. These local delivery strategies, including collagen membranes, are posited as significant advancements in enhancing therapeutic outcomes for glioblastoma patients.

## 1. Introduction

Glioblastoma is classified as one of the most aggressive brain malignancies, with an average overall survival rate post-diagnosis of approximately 15 months, even when subjected to existing therapeutic regimes [1]. The established clinical regimen, referred to as the Stupp protocol, consists of maximal surgical resection when possible, supplemented by radiotherapy and adjuvant temozolomide chemotherapy [1]. Yet, due to the propensity of glioblastoma cells to pervade adjacent healthy brain tissue, patients frequently experience lethal tumor recurrences [2].

The quest for innovative therapeutic approaches is impeded by several hurdles, including the intrinsic heterogeneity of the tumor, the invasive nature of the cancer cells, resistance to alkylating chemotherapeutic agents, and formidable physiological barriers. Currently, the therapeutic arsenal available in the clinical market is limited. A particularly notable approach within the current paradigm involves localized therapy, which aligns with the invasive nature of tumor resection already embedded within the standard of care [3].

In the landscape of localized treatments, the utilization of Gliadel^®^ wafers, which are administered intraoperatively, has been distinguished. Despite their introduction into clinical practice, these wafers have been associated with considerable adverse effects and have not substantially extended therapeutic outcomes, although the concept of local drug delivery remains an appealing strategy [4].

The employment of biodegradable/bioresorbable membranes for the administration of chemotherapeutic agents in the treatment of high-grade gliomas presents several advantages. The localization of drug release directly into the tumor bed is critical, especially considering that recurrences typically occur within 2 cm of the resection margin. This targeted approach facilitates the use of reduced drug dosages in comparison to systemic intravenous methods, thereby mitigating the risk of systemic adverse effects [5]. Enhanced bioavailability of the drug at the site of the tumor is another significant benefit, with the added capability of modulating the release kinetics of the drug, including options for sustained or staged release [5]. Such improvements in therapeutic outcomes have been attributed to local delivery methods utilizing polymers, gels, and micro- and nanoparticle technologies [6].

Collagen membranes show bioresorbability, meaning they degrade and are absorbed by the body, which is crucial for medical applications to avoid long-term foreign material presence. Collagen, unlike gelatin, maintains structural integrity and stability, making it ideal for supporting tissue regeneration.

Synthetic polymers, while biodegradable, often raise biocompatibility concerns and may produce toxic degradation products. Collagen closely mimics the extracellular matrix, promoting cell growth and integration, and its proven success in clinical settings like in Gliadel supports its selection. This choice ensures the membranes are safe, effective, and aligned with the requirements for biomedical use.

Irinotecan, also known as irinotecan hydrochloride trihydrate or CPT-11, is used in these biodegradable implants and acts as a topoisomerase I inhibitor. This water-soluble, semi-synthetic derivative of the plant alkaloid camphothecin exerts its effect by binding to the DNA-topoisomerase I complex, hindering the re-ligation of single-strand breaks introduced by the enzyme during DNA replication, consequently inducing double-stranded DNA breaks and cell apoptosis. Although the contribution of its active metabolite, SN-38, in human therapy is not fully delineated, it is acknowledged that irinotecan’s efficacy is cell cycle-specific, targeting the S phase [7]. Despite its capacity to penetrate the blood–brain barrier, therapeutic concentrations in cerebral tissue necessitate intravenous administration at doses ranging from 125 to 500 mg/m^2^, which is often associated with substantial systemic toxicity, including severe neutropenia, gastrointestinal complications, and diarrhea [8].

Minocycline, incorporated into biodegradable implants, is characterized by distinct pharmacodynamic attributes and belongs to the tetracycline pharmacotherapeutic class, bearing the Anatomical Therapeutic Chemical (ATC) classification code J01AA08. The formulation Minoz MR comprises the active compound minocycline as minocycline hydrochloride, a semi-synthetic tetracycline derivative. Minocycline distinguishes itself from other tetracyclines by exhibiting enhanced efficacy against a range of microorganisms, including *Staphylococcus aureus*, *Nocardia* spp., *Propionibacterium acnes*, *Neisseria meningitidis*, *Neisseria gonorrhoeae*, certain *Streptococcus* species, some *Enterobacteriaceae*, *Acinetobacter*, Bacteroides, *Haemophilus*, and specific mycobacteria like *M. leprae* [9,10].

As a bacteriostatic antibiotic, minocycline’s spectrum of action is extensive, effectively targeting various pathogenic microorganisms [11]. Regarding the mechanism of action, after penetration in bacterial cells, the antibiotic molecules reversibly bind to the 30S ribosomal subunit. This interaction disrupts the attachment of aminoacyl-tRNA to the mRNA–ribosome complex, impeding bacterial protein synthesis and bacterial growth inhibition [11]. Furthermore, minocycline has been observed to inhibit the activation of the MAP kinase p38 in microglia and suppress the secretion of chemokines, mechanisms that are implicated in glial cell-induced expression of type 1 matrix metalloprotease (MT1-MMP) in tumor-associated microglia. The microglia leverage MT1-MMP to facilitate invasion through matrix degradation. Excessive MT1-MMP expression by microglia is associated not only with glioma invasion but also with the revascularization of tumors. Minocycline’s role extends to inhibiting the upregulation and enzymatic activity of MT1-MMP in microglia stimulated by glioma-conditioned media. In the context of glioma therapy, Markovik and colleagues have presented evidence suggesting that minocycline attenuates the pro-tumorigenic properties of glioma-associated microglia, positioning it as a potential adjunct to existing treatment strategies for patients with gliomas [12,13,14].

Thus, it can be concluded that both active substances, minocycline and irinotecan, are beneficial for local delivery therapy in glioblastoma due to their ability to inhibit tumor growth and reduce inflammation at the site of the tumor. Therefore, minocycline and irinotecan were explored for local delivery therapy in glioblastoma due to their potential benefits:

- Minocycline: This antibiotic has shown promise in glioblastoma treatment due to its ability to inhibit matrix metalloproteinases (MMPs) and reduce inflammation. MMPs play a role in tumor invasion and angiogenesis, so inhibiting them can help slow down the progression of glioblastoma. Minocycline also has neuroprotective properties, which could help preserve surrounding healthy brain tissue during treatment [13,15].
- Irinotecan: This chemotherapy drug works by inhibiting topoisomerase I, an enzyme involved in DNA replication and repair. By interfering with DNA processes, irinotecan can cause cell death in rapidly dividing cancer cells. In glioblastoma, where cells proliferate quickly, irinotecan can be effective in slowing down tumor growth [16].

When delivered locally, these drugs can achieve higher concentrations at the tumor site while minimizing systemic side effects. Local delivery methods such as convection-enhanced delivery (CED) or drug-eluting implants allow for targeted administration directly into the tumor or surrounding tissue, maximizing therapeutic efficacy [17].

The optimal therapeutic strategy for Glioblastoma Multiforme (GBM) involves extensive surgical resection complemented by the Stupp protocol, a regimen instituted in 2005 that integrates concurrent and subsequent radiotherapy with chemotherapy. Following this protocol correlates with a median survival period of 15 months [15,18].

The significant improvement in survival rates, observed between the patients following the Stupp protocol and those undergoing radiotherapy alone [19], underlines the efficacy of the multimodal treatment, affirming the importance of surgical resection followed by coordinated chemoradiation therapy in the treatment paradigm for GBM.

The predominant factor contributing to the ineffectiveness of many glioblastoma therapies is attributed to the systemic administration of chemotherapeutic agents, either intravenously or orally. The central challenge presented by this approach is the impediment of the blood–brain barrier (BBB) [20], a selective permeability barrier that favors the passage of small, nonpolar, and lipophilic molecules, thereby facilitating their transit into the brain [21]. In contrast, the majority of chemotherapeutic drugs are characterized by large, hydrophilic structures and carry ionic charges, which impede their ability to infiltrate the BBB effectively. To achieve therapeutic concentrations within the brain, elevated systemic doses are often required, which can lead to increased systemic toxicity [22].

Systemic administration also suffers from the rapid diffusion of drugs once they have crossed the BBB, making it difficult to sustain therapeutic concentrations within the cerebral tissue. Local delivery methods circumvent these issues by directly administering the chemotherapeutic agent into the tumor bed, which enhances bioavailability at the target site, decreases the required dosage, and reduces systemic side effects [22].

Local delivery is deemed particularly advantageous for glioblastoma, given that recurrences predominantly occur within a 2 cm radius of the original resection site. Thus, various local delivery modalities, including polymer-based systems, gels, and micro- and nanoparticle formulations, are under examination. These strategies enable direct application to the parenchyma of the resected cavity and have been associated with a notable enhancement in life expectancy, yielding a 25% increase [23].

The objective of this study is to explore and advance novel implant-based therapeutic interventions for glioblastoma. Employment of biodegradable/bioresorbable membranes which are doped with alkylating agents could represent a treatment avenue for gliomas [24]. Furthermore, this manuscript suggests that synergizing this local treatment modality with broader targeting strategies that encompass the tumor microenvironment, in accordance with the Stupp protocol, may offer a pathway to enhance clinical outcomes in glioblastoma management [25].

## 2. Materials and Methods

### 2.1. Materials

Extraction of type I fibrillar collagen from bovine hide (from local slaughter house, (Domidene SRL, Posesti, Romania)) was accomplished through fibril-forming technology at the National Institute for Research and Development in Textiles and Leather, Bucharest. The bovine dermis was treated with an alkaline solution containing 5–10% sodium hydroxide and 1 M sodium sulfate at ambient temperature over a period of two days. This was succeeded by an acidic treatment with 1 N HCl to achieve full solubilization of the collagen in its undenatured state. Following this, salt precipitation and subsequent resolubilization were performed to purify the collagen, resulting in a gel concentration of 2.71% (*w*/*v*), quantified through gravimetric analysis after drying at 105 °C.

The utilized glutaraldehyde was a 2.5% aqueous solution (Alfa Aesar, Haverhill, MA, USA), selected for its capacity to crosslink and stabilize the collagen gel, creating a non-toxic compound for human tissue applications [26]. Both the minocycline hydrochloride trihydrate and irinotecan hydrochloride trihydrate were ACROS ORGANICS products, with a purity of 97%.

### 2.2. Obtainment of Membranes

The manufacture of the biodegradable membranes consisted in mixing 2.71% collagen gel derived from bovine derma with glycerin and distilled water. The pH of the obtained solution was adjusted to 7.2–7.4 using 1 M sodium hydroxide. At the end, glutaraldehyde solution was added to facilitate collagen crosslinking. The choice of crosslinking agent was due to the fact that glutaraldehyde is renowned for its crosslinking efficacy, inducing reactions between its aldehyde groups and the free amine groups of lysine or hydroxylysine within the polypeptide chains.

Drug-based membranes were fabricated in a similar way. Firstly, the active substance (irinotecan or minocycline) was dissolved in water. Then, a specific amount of collagen gel was mixed with glycerin and adequate volumes of irinotecan or minocycline solutions. The pH of the resulting solution was adjusted to 7.2–7.4 with 1 M sodium hydroxide, and finally, the crosslinking agent, glutaraldehyde, was added. The membranes were formulated to contain irinotecan in five varying concentrations of the chemotherapeutic agent relative to the pure collagen—10%, 20%, 30%, 40%, and 50%—or minocycline in two different concentrations—40% and 20%—relative to the pure collagen.

The irinotecan concentrations were chosen based on Gawley’s study [17], which investigated implantable drug-eluting systems (iDESs) containing different concentrations of irinotecan (10–50% *w*/*w*) and various lengths (2, 3, and 6 mm), tested in a mouse model of glioblastoma resection, or considered for potential clinical trials based on tumor depth. The study compared the cytotoxicity of irinotecan to temozolomide (TMZ) against the tumor border (BAT) in patients, revealing significantly higher efficacy (*p* = 0.01) with irinotecan, reducing cell viability below 50% in all samples. IRN had a response rate of 75% and reduced cell viabilities to below 45% and in some to as low as 10%. Based on these data, IRN is clearly a more effective drug for treating recurrent GBM when administered directly to the cancer cells [27]. 

The minocycline doses utilized in our study were informed by the specialized literature, including studies by Giuliani et al. [28] and others [15,29]. These studies explored the in vivo efficacy of intracranial minocycline delivered via a biodegradable controlled-release polymer against rat intracranial 9 L gliosarcoma. The incorporation of minocycline into the biodegradable polymer polyanhydride poly[bis(p-carboxyphenoxy)propane-sebacic acid] (pCPP:SA) at a ratio of 50:50 by weight was investigated to assess its potential synergistic effects with systemic 1,3-bis (2-chloroethyl)-1-nitrosourea (BCNU).

These solutions were then transferred into Teflon Petri dishes for subsequent desiccation at ambient temperature in order to obtain the membranes.

**Figure 1:**
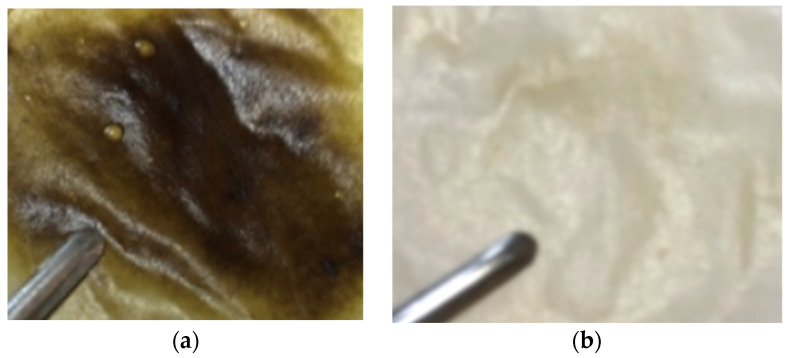
Collagen membranes with minocycline 40% (**a**) and irinotecan 10% (**b**).

Table 1 shows the membranes’ compositions, while Figure 1 presents an example of the obtained membranes. For each case, 3 membranes were obtained which were further tested.

Conclusions drawn from the experimental trial indicate that minocycline-based membranes displayed a propensity for slight fragility and elasticity.

### 2.3. Methods

#### 2.3.1. Water Absorption Tests

Water absorption tests were conducted on collagen membranes submerged in a PBS buffer solution of 7.4 at 25 degrees Celsius. The water uptake was quantified at various time points, including 5, 10, 15, 20, and 30 min, and 1, 2, 3, 4, 5, 6, 7, 8, and 23 h. The calculation employed the following formula for percentage water absorption:

$$Water\;absorption=\frac{w-w_{0}}{w_{0}}\times 100$$

where *w*_0_ denotes the dry weight and *w* the wet weight. Each assay was performed in triplicate.

#### 2.3.2. Enzymatic Degradation

The enzymatic degradation test was conducted using collagenase from *Clostridium histolyticum*, sourced from Sigma-Aldrich (St. Louis, MO, USA), and a phosphate-buffered saline (PBS) solution with a pH of 7.4. Membranes of a pre-established weight were submerged in the PBS solution and incubated at 37 °C overnight. Collagenase (10 μg/mL) was then added, and the test tube was replaced at 37 °C. At various time intervals, the degradation process was halted by removing the membrane from the degradation solution, followed by compression and reweighting. The percentage of mass lost was calculated as follows:

$$\%\;Collagen\;Mass\;Degraded=\frac{w_{i}-w_{t}}{w_{i}}\times 100$$

where *w_i_* represents the initial mass and *w_t_* denotes the mass after a time *t*. Each test was performed in triplicate.

Each biomaterial was obtained in parallel, in three copies, and each material was further tested. The resulting graphs (water absorption and enzymatic degradation) were made considering the arithmetic mean of the measurements obtained for each specimen, with the corresponding standard error. The data obtained were then statistically analyzed with the Graph Pad Prism 5 program, in which the values were compared with the negative control, considered in this case to be the collagen membrane, for both water absorption and enzymatic degradation studies. The statistical significance of the obtained results was determined according to the value of the parameter “*p*” generated by the statistical program, which is represented on the graph according to its value, by means of *, **, or *** notations.

#### 2.3.3. Antimicrobial Test

The antimicrobial effects were tested using the Kirby Bauer diffusive disks method on Mueller Hinton culture media, with agar, using Petri plates with a diameter of 50 mm, inoculated with gram-negative microorganisms, type *Escherichia coli*, or gram-positive type *Staphylococcus aureus* according to the 0.5 McFarland standard. Each bacterial inoculum used was 24 h old [30,31].

After inoculation, the plates were incubated for 18–24 h at 37 °C. After incubation, the inhibition diameters achieved were measured with a ruler. For a better visualization of inhibition diameters, when needed, a colony reader was used.

The results obtained were compared with the results obtained for minocycline, which was tested in the form of a solution with a concentration of 5 mg/mL (solution made in DMSO 20%). The diffusive disks used in the minocycline tests were 6 mm in diameter. All tests were performed in triplicates, and the results are presented as average inhibition diameter ± standard deviation.

#### 2.3.4. In Vitro Drug Release

The in vitro drug delivery characteristics incorporated in the engineered membranes were evaluated using a paddle dissolution apparatus equipped with a sandwich device as detailed in our previous works [32,33]. The content of each drug in the samples extracted from the release vessels at different time intervals was determined spectrophotometrically at a wavelength of 246 nm for minocycline and 255 nm for irinotecan, respectively, corresponding to their maximum absorbance, using the curve standard calibration. Each test was performed in triplicate. Kinetic patterns plotted as the cumulative amount of drug released (%) versus time were recorded.

## 3. Results and Discussion

Collagen membranes, endowed with precise physical, chemical, and biological properties, have attracted significant interest in the field of tissue engineering and drug delivery systems. The present study engaged in crosslinking type I collagen gels with glutaraldehyde, incorporating both minocycline and irinotecan, which function as antibiotic and chemotherapeutic agents, respectively.

### 3.1. Water Absorption Test

The rationale behind conducting water absorption testing of the membranes is to analyze their interaction with the environment, bioviability, which shapes their biodegradability properties. These aspects directly influence drug release. As long as the membrane interacts with the environment, it absorbs water—in the present case, cerebrospinal fluid, which contains 99% water—causing it to expand and break conjugated bonds, leading to drug release and membrane biodegradation. We are interested in the absorption capacity to determine its behavior. If a membrane does not absorb water, this indicates a lack of interaction with the environment, suggesting a lack of biodegradability properties.

Also, the water absorption test for biodegradable membranes loaded with substances such as irinotecan or minocycline evaluates their ability to absorb and retain drugs. This helps determine their effectiveness in controlled drug release, crucial for applications such as cancer treatment or infection control.

**Figure 2:**
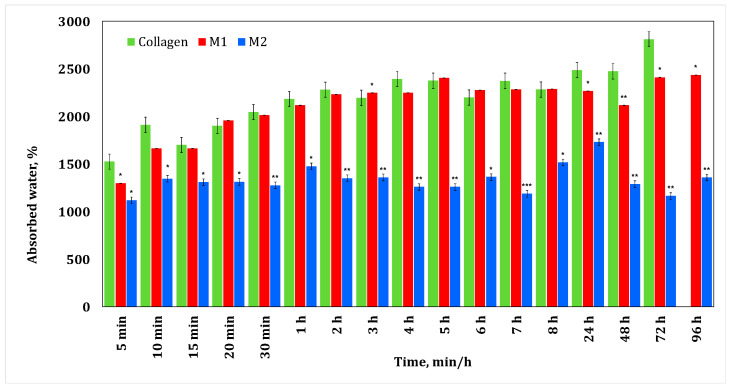
Water absorption for collagen membranes with different concentrations of minocycline. A *p*-value > 0.5 indicates that the results obtained are not statistically significant. “*” corresponds to results with statistical significance (0.5 < *p* < 0.1), “**” corresponds to distinctly statistically significant results (0.1 ≤ *p* < 0.01), and “***” corresponds to highly statistically significant results (*p* ≤ 0.01).

**Figure 3:**
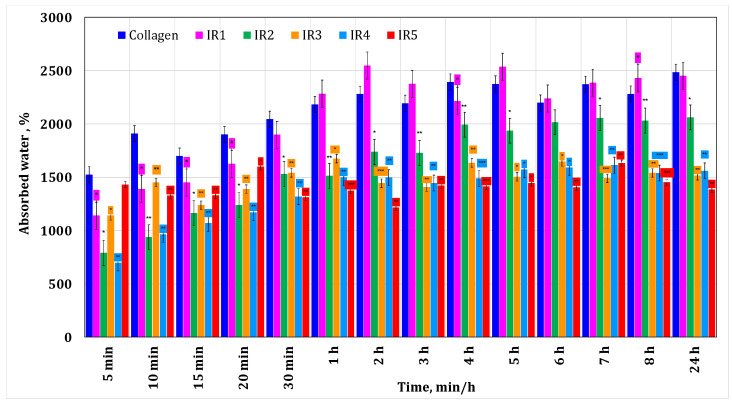
Water absorption for the collagen-based membranes with different irinotecan concentrations. A *p*-value > 0.5 indicates that the results obtained are not statistically significant. “*” corresponds to results with statistical significance (0.5 < *p* < 0.1), “**” corresponds to distinctly statistically significant results (0.1 ≤ *p* < 0.01), and “***” corresponds to highly statistically significant results (*p* ≤ 0.01).

Figure 2 shows the results obtained during the water absorption tests for the minocycline-doped membranes. It can be seen that a low concentration of minocycline has no influence on collagen water absorption, while a high concentration of minocycline results in a decrease in water absorption compared to the behavior of the control collagen membrane. This could suggest that during membrane formation, minocycline interacts with collagen. Similar behavior was observed for irinotecan-based membranes (Figure 3). In both cases, the maximum water absorption is about 2500%.

Typically, a glioblastoma tumor is approximately 4 cm [34]. After the surgical resection, the resulting cavity can be filled with a membrane of a maximum of the same size. The fact that this membrane retains water could be beneficial for treatment management, knowing that cerebrospinal fluid contains 99% water [34].

### 3.2. Enzymatic Degradation

The enzymatic degradation test for a biodegradable membrane loaded with irinotecan or minocycline aims to evaluate how the membrane degrades in the presence of specific enzymes found in the body, such as those in brain tissue or other relevant biological environments. This test is crucial for determining the rate and manner of degradation of biodegradable membranes and how they release the incorporated active substances.

The main roles of the enzymatic degradation assay are simulating physiological conditions, evaluating the degradation rate, determining the drug release efficiency, and studying the degradation behavior. Some proteolytic enzymes are able to reversibly increase the permeability of the blood–brain barrier (BBB); collagen in the basement membrane plays a role in regulating the permeability of the BBB [29]. The enzyme collagenase has an effect on the permeability of the BBB and on the structure of the extracellular matrix (ECM) [35].

**Figure 4:**
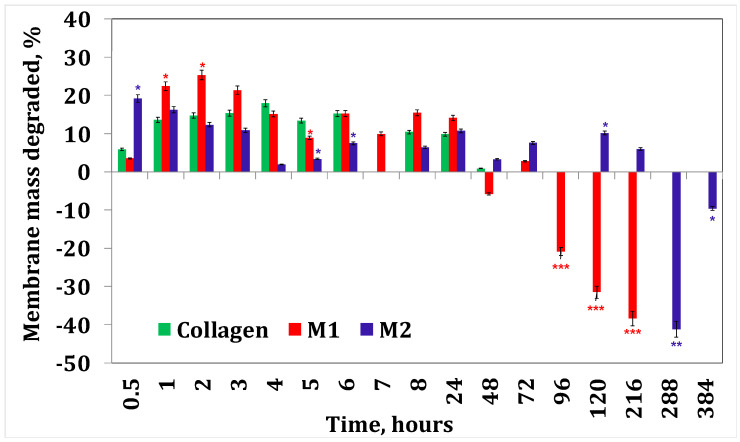
Collagenase degradation of collagen membranes with minocycline. A *p*-value > 0.5 indicates that the results obtained are not statistically significant. “*” corresponds to results with statistical significance (0.5 < *p* < 0.1), “**” corresponds to distinctly statistically significant results (0.1 ≤ *p* < 0.01), and “***” corresponds to highly statistically significant results (*p* ≤ 0.01).

**Figure 5:**
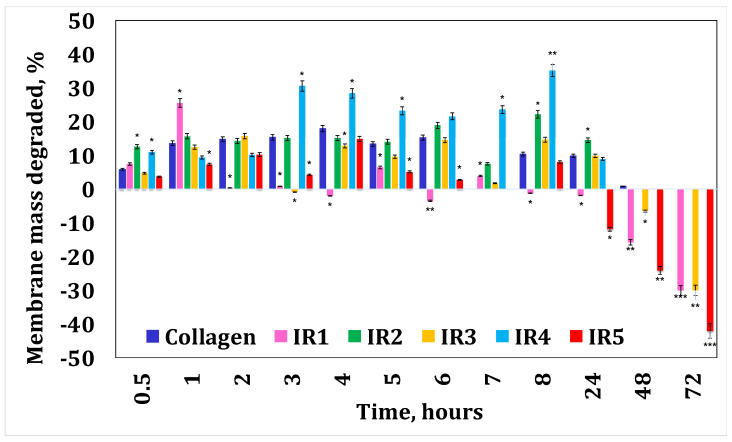
Collagenase degradation of the collagen membranes with irinotecan. A *p*-value > 0.5 indicates that the results obtained are not statistically significant. “*” corresponds to results with statistical significance (0.5 < *p* < 0.1), “**” corresponds to distinctly statistically significant results (0.1 ≤ *p* < 0.01), and “***” corresponds to highly statistically significant results (*p* ≤ 0.01).

Figure 4 and Figure 5 present the results obtained for enzymatic degradation. It can be seen that if the membrane is initially degraded, after 4 h this degradation is stopped and the membrane starts to absorb liquid. These results confirm that minocycline released by the membrane inhibits the enzyme [36], and at the same time they also suggest that irinotecan released by the membrane inhibits the enzyme after 24 h. On the other hand, from the water absorption tests, a significant amount of water absorbed can be observed, hence it can be stated that after 24 h there could also be competition between the water absorption process and enzymatic degradation. However, these membranes finally disintegrate.

### 3.3. Antimicrobial Tests

The common infections associated with glioblastoma excision surgeries, such as postoperative meningitis, brain abscess [37], and surgical site infections [38,39], are often caused by bacteria such as the following:

- *Staphylococcus aureus*: A bacterium that can cause a variety of infections, including surgical site infections and meningitis;
- Streptococcus species: Particularly *Streptococcus pneumoniae* and *Streptococcus pyogenes*, which can cause meningitis and brain abscesses;
- *Escherichia coli* (*E. coli*): Known for causing surgical site infections and urinary tract infections, but can also contribute to postoperative meningitis in certain cases;
- *Pseudomonas aeruginosa*: An opportunistic pathogen that can cause various infections, including surgical site infections and meningitis, especially in immunocompromised individuals.

The relevance of testing the antimicrobial potential of a degradable membrane in the context of using it as a treatment after surgical resection of glioblastoma lies in ensuring that the membrane effectively prevents bacterial colonization and infection at the surgical site. By assessing its ability to inhibit the growth of common pathogens like *Staphylococcus aureus* and *E. coli*, researchers can determine if the membrane could serve as a protective barrier against postoperative infections, thus promoting better patient outcomes.

**Figure 6:**
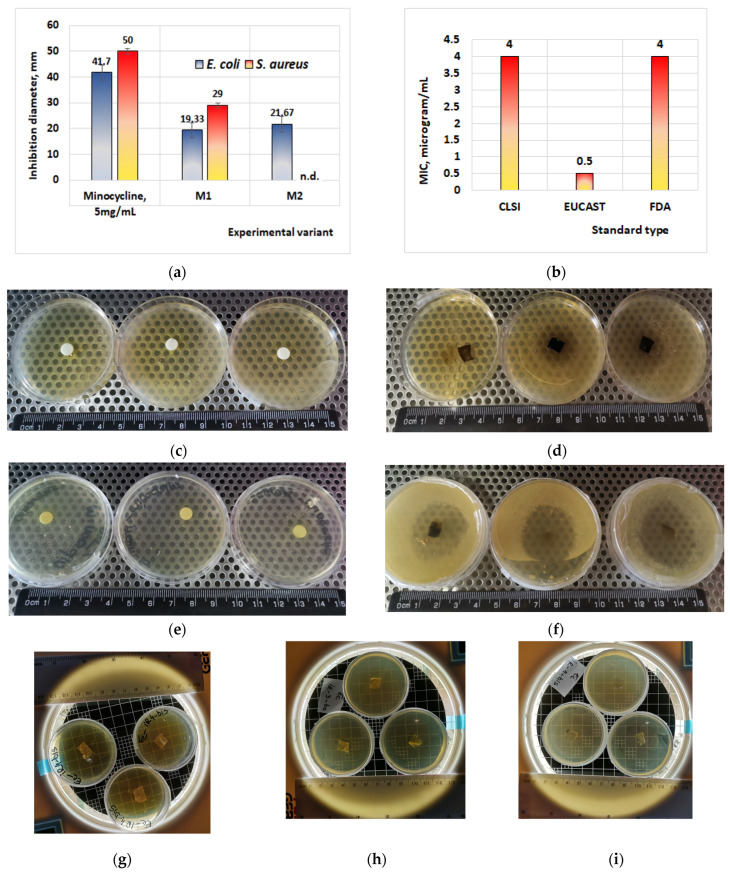
Antibacterial effects of minocycline. (**a**) Test performed on thin biofilm; (**b**) minimal inhibitory concentration of minocycline on *S. aureus*, for different applied standards (data adapted after Bidell et al., 2021 [44]); (**c**) effect of minocycline 5 mg/mL on *E. coli*; (**d**) effect of thin film M1 on *E. coli*; (**e**) effect of minocycline 5 mg/mL on *S. aureus*; (**f**) effect of thin film M1 on *S. aureus*; (**g**) effect of thin film IR4 with irinotecan on *E. coli*; (**h**) effect of thin film IR3 with irinotecan on *E. coli*; (**i**) effect of thin film IR1 with irinotecan on *E. coli*. n.d. = not determined; CLSI = Clinical and Laboratory Standard Institute; EUCAST = European Committee on Antimicrobial Susceptibility; FDA = Food and Drug Administration, USA.

The results obtained in this study on the antibacterial effects (Figure 6a–f) showed that the best results are obtained in the case of *S. aureus* (microorganism G+) when inhibition diameters of 50 mm are obtained in the case of using disks impregnated with a solution containing 5 mg minocycline/mL and diameters of 29 mm are obtained in the case of membranes with minocycline (Figure 6a,e,f).

According to the current standards [40] regarding the minimum inhibitory concentration of *S. aureus* for minocycline (Figure 6b) (EUCAST, FDA, or CLSI), there is an inverse proportionality between the value of the minimum inhibitory concentration (MIC) and the diameter of inhibition. Therefore, a large value for the inhibition diameter corresponds to a small value for the MIC. If for the sample M1 (20% minocycline) we obtain an inhibition diameter of 29 mm, then for the material M2 (40% minocycline) the inhibition diameter will be larger compared to M1. The corresponding MIC values for the material will probably decrease in the following order: MIC value of M1 > MIC value of M2.

Antimicrobial effects were also obtained in the case of *E. coli* (microorganism G−), when inhibition diameters of 41.7 mm were obtained in the case of disks impregnated with 5 mg/mL minocycline solution (Figure 6a,c). In the case of films with minocycline, the inhibition diameters obtained are between 19.33 and 21.67 mm (Figure 6a,d), with the inhibition diameters obtained being probably directly proportional to the concentration of the minocycline from the membrane.

The antimicrobial tests carried out on membranes containing only irinotecan showed that they have no antimicrobial effect (Figure 6g–i), because neither irinotecan nor other components of the produced membranes (collagen and glycerol) exhibit antibacterial properties.

The conclusions obtained in the case of minocycline in the present study are also confirmed in other international standards (Figure 6b) and in the specialized literature. Thus, the in vitro studies performed by Chopra et al. [41] on *S. aureus* MRSA demonstrated that when treating biofilms generated in wells of *S. aureus* MRSA (each well inoculated with 10^8^ CFU/mL), with 100 µL minocycline/well (i.e., 4 µg/mL), the bacterial population is reduced from 10^8^ to 10^0.04^ in one-day-old biofilms.

The determinations made by Chopra et al. on biofilms of different ages showed that for older biofilms, a greater degree of reduction in the bacterial population occurred. Thus, when 7-day-old biofilms are treated with minocycline, the bacterial population is reduced to values between 10^0.29^ and 10^0.08^ [41].

The tests performed by Phair et al. [42] showed that chemoresistant *S. aureus* strains isolated from patients were found to be susceptible to minocycline, at MIC concentrations (MBC) ranging from 0.05 (0.1) to 1.56 (3.12) mg/mL. Lewis et al. [43], in studies carried out on 983 *S. aureus* strains isolated from patients, found that only 0.4% of them were resistant to minocycline.

Regarding the degree of metabolism, biochemical tests performed on patients who received 100 mg minocycline/day showed that in the blood plasma the maximum concentration of minocycline found was 4.1 mg/mL, which suggests a bioavailability of approximately 90–100% [44] for a maximum oral dose of 100 mg/every 12 h or 200 mg every 24 h [40]. Because it is liposoluble, minocycline easily crosses the blood–brain barrier and can be successfully used in the treatment of neuro-infections with *S. aureus* [45,46]. In addition to the antibacterial effect, minocycline can inhibit the chronic activation of microglial cells (microglial cells or microglia = resident immune cells of the central nervous system, [47]), a fact that recommends it in the treatment of neurodegenerative processes.

One such study, conducted by Strickland et al. [48] on a group of 15 patients with head trauma who were administered minocycline, demonstrated that, in addition to the antimicrobial effect, it significantly reduces the process of chronic activation of microglial cells. In addition, studies conducted by the same scientists on a group of 74 patients with ischemic stroke, who were given 200 mg of minocycline per day for 5 days, showed that these patients improved their neurological functions after an interval of 7–30 days [44]. Other scientists reported that neurological examination of 30 patients with spinal cord injuries, who were given prednisolone (30 mg) and minocycline (50 mg) every 12 h, showed that after 6 months, 40% of the motor functions of the patients remained functional and 7.4% of the patients fully recovered their motor functions [49].

### 3.4. In Vitro Drug Release

**Figure 7:**
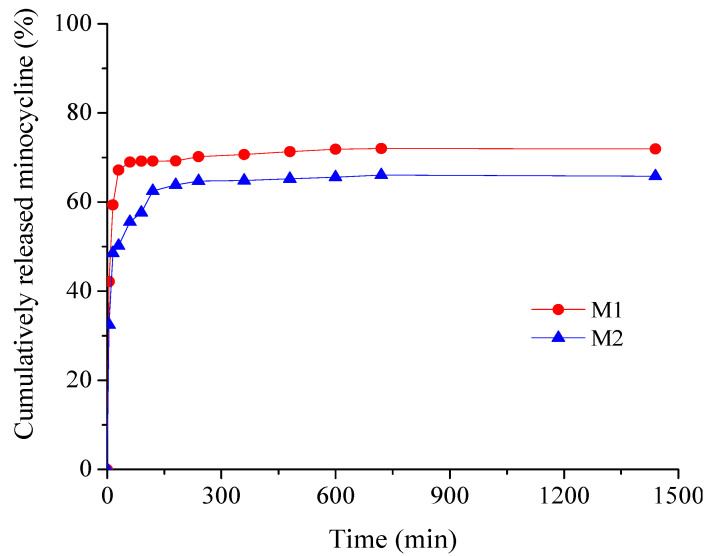
Time-dependent cumulative release patterns of minocycline from collagen membranes.

**Figure 8:**
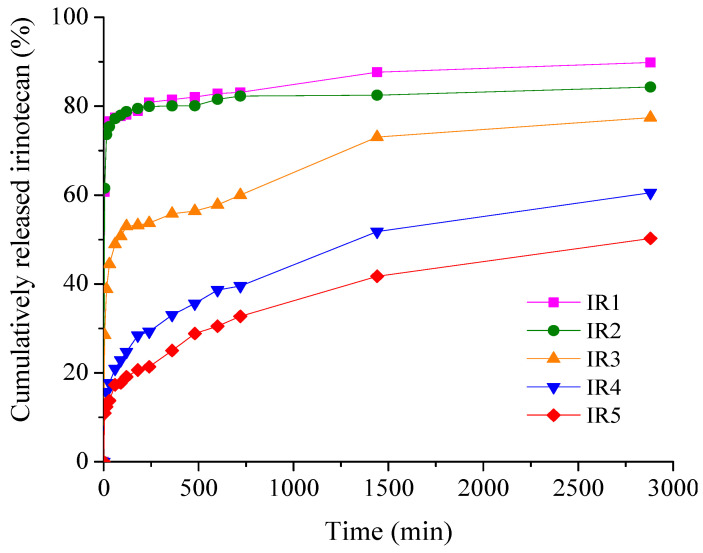
Time-dependent cumulative release patterns of irinotecan from collagen membranes.

Antibiotic and chemotherapeutic delivery patterns plotted as cumulative percent drug released versus time are shown in Figure 7 for minocycline membranes and Figure 8 for irinotecan membranes, respectively.

Drug release is investigated through the influence of drug concentration on kinetic profiles. As can be seen from Figure 7, minocycline samples show similar profiles with an obvious burst release effect in the first 15 min (48.57% for M2 and 59.35 for M1), beneficial for ensuring an aseptic environment in the immediate postoperative period, followed by a sustained drug release for up to 24 h to prevent further bacterial invasion or multiplication [32,50]. The cumulative amount of minocycline released (%) over 24 h was 71.95% for the low drug concentration sample M1 and 65.81% for the high drug concentration sample M2.

From Figure 8, it can be seen that for the samples with irinotecan, the burst release effect in the first 15 min is more pronounced for the low drug concentration samples, 74.45% (IR1) and 73.61% (IR2), while for the membranes with higher drug loading, there was a significant decrease, about 2 times for IR3 and about 5–6 times for IR4 and IR5, respectively. After 48 h of experiments, the cumulative drug release percentages ranged from 50.25% (IR5) to 89.85% (IR1). The high local concentration of irinotecan released in a short period of time ensures the remission of tumor cells, while in the following hours the release of the drug is gradual and sustained, ensuring adequate concentrations to consolidate the remission and cure the tumor [51].

The release behavior is well correlated with the data obtained from the water absorption study, with significant water uptake being recorded for both minocycline-based and irinotecan-based collagen membranes.

## 4. Conclusions

A recent study published in February 2024 demonstrates that implants are a promising new treatment for glioblastoma that could be rapidly translated into the clinic [52]. We can state that in the particular case of glioblastoma, nanoengineering and sonodynamic therapy have proven to be good options; therefore, the proposed collagen-based membranes represent an exhilarating therapeutic opportunity for treating brain cancers that show a difficult response to drugs.

However, membranes using minocycline and irinotecan have not yet been created in response to high-grade glioma, yet these two substances represent good therapeutic agents for this type of malignancy. The results obtained in this study revealed that the type and concentration of the drug strongly influence the water absorption, enzyme degradation, antimicrobial activity, and delivery characteristics of the designed membranes, and for each type of membrane the biphasic allure of the kinetic profiles is favorable for local prophylaxis and treatment in glioblastoma. The drug delivery results obtained suggest that the membrane containing 30–50% irinotecan continues to release the drug even after 2 days, while the membrane with 40% minocycline ensures substantial drug concentrations during the first 5 h and thereafter provides lower concentrations even after 24 h. The slow release of irinotecan ensures a constant and effective therapeutic concentration of the drug, which can lead to the maximization of the therapeutic effect, reducing the risk of tumor recurrence. Thus, these membranes could be effective in local treatment due to the better balance of the burst release effect with prolonged delivery in the case of the antibiotic and the prolonged presence of the local anticancer chemotherapeutic drug. As a result, the development of these membranes represents a step forward in the development of a treatment for the dreaded disease that is brain cancer. In this regard, more medical or clinical studies should be conducted to establish the limits and possible clinical implementation of these membranes.

## Figures and Tables

**Table 1:** Tested membranes and their composition.

Sample Code	Minocycline (%)	Irinotecan (%)
C	0	0
M1	20	-
M2	40	
IR1	-	10
IR2	-	20
IR3	-	30
IR4	-	40
IR5	-	50

## Data Availability

The original contributions presented in this study are included in this article; further inquiries can be directed to the corresponding author.
